# Multispectral optoacoustic tomography for non-invasive disease phenotyping in pediatric spinal muscular atrophy patients

**DOI:** 10.1016/j.pacs.2021.100315

**Published:** 2021-11-10

**Authors:** Adrian P. Regensburger, Alexandra L. Wagner, Vera Danko, Jörg Jüngert, Anna Federle, Daniel Klett, Stephanie Schuessler, Adrian Buehler, Markus F. Neurath, Andreas Roos, Hanns Lochmüller, Joachim Woelfle, Regina Trollmann, Maximilian J. Waldner, Ferdinand Knieling

**Affiliations:** a Department of Pediatrics and Adolescent Medicine, Friedrich-Alexander-University (FAU) Erlangen-Nuremberg, Erlangen, Germany; b Medical Department 1, Friedrich-Alexander-University (FAU) Erlangen-Nuremberg, Erlangen, Germany; c Department of Pediatric Neurology, Developmental Neurology and Social Pediatrics, University of Duisburg-Essen, Essen, Germany; d Brain and Mind Research Institute, University of Ottawa, Ottawa, Canada; e Division of Neurology, Department of Medicine, The Ottawa Hospital, Canada; f Children's Hospital of Eastern Ontario Research Institute, University of Ottawa, Ottawa, Canada

**Keywords:** Optoacoustics, Photoacoustics, Multispectral optoacoustic tomography, Spinal muscular atrophie

## Abstract

Proximal spinal muscular atrophy (SMA) is a rare progressive, life limiting genetic motor neuron disease. While promising causal therapies are available, meaningful prognostic biomarkers for therapeutic monitoring are missing. We demonstrate handheld Multispectral Optoacoustic Tomography (MSOT) as a novel non-invasive imaging approach to visualize and quantify muscle wasting in pediatric SMA. While MSOT signals were distributed homogeneously in muscles of healthy volunteers (HVs), SMA patients showed moth-eaten optoacoustic signal patterns. Further signal quantification revealed greatest differences between groups at the isosbestic point for hemoglobin (SWL 800 nm). The SWL 800 nm signal intensities further correlated with clinical phenotype tested by standard motor outcome measures. Therefore, handheld MSOT could enable non-invasive assessment of disease burden in SMA patients.

## 1 Introduction

Spinal muscular atrophy (SMA) is a severe neuromuscular disease caused by a homozygous deletion or mutation in the survival motor neuron 1 gene on chromosome 5q, resulting in insufficient expression of the survival motor neuron (SMN) protein ^1^. This leads to the degeneration of motor neurons in the spinal cord and brain stem with consecutive muscular atrophy and weakness of skeletal muscles [1], [2]. A closely related gene, survival motor neuron 2 (SMN2), also produces the SMN protein, partially compensating the loss of SMN1 by SMN2 protein synthesis [3]. Individuals with a higher copy number of SMN2 do in general have a milder phenotype [4], [5]. However, exact clinical subtypes (SMA1–4) are defined by disease onset, its severity as well as by its hereditary and distribution patterns [6], [7]. Newborns diagnosed with SMA type 1 never achieve the ability to sit, and in this age group, SMA is the most common genetic cause of death [8], [9], [10]. The approval of the first causal intrathecal treatment to restore the SMN protein expression was an important milestone in SMA treatment regimens [11], [12]. Furthermore, therapeutic modulating of SMN2 gene splicing [13] and SMN1 AAV9-gene replacement [14], [15], [16] are now broadening the therapeutic horizon for these patients. While great attention was drawn to the development of novel drug therapies, prospective monitoring modalities and candidate biomarkers, such as cerebrospinal fluid neurofilaments or quantitative magnetic resonance imaging (MRI), to objectively assess the extent of disease or response to therapy are not satisfactory and difficult to implement [17], [18], [19]. In clinical practice, newborns with muscular hypotonia are at high risk for complications during intravenous anesthesia and consecutive respiratory insufficiency. Besides the individual risk factors, the staff requirements are correspondingly high. Therefore, disease progression and treatment success are assessed using validated motor function tests and development scales [20]. These tests might be affected by general development gains, learning effects, daytime tiredness or they might be unsuitable for assessing very young subjects. All of this might potentially lead to inaccuracy in disease and treatment monitoring. Accordingly, there is an unmet urgent clinical need – especially among presymptomatic and very young patients – for non-invasive technologies that enable rapid and objective assessment of the disease state and progression with the lowest burden possible. Multispectral Optoacoustic Tomography (MSOT) is a laser-based radiation free, bedside imaging technology harbouring the ability to non-invasively visualize and quantify endogenous tissue compounds such as haemoglobin, lipids, and collagen [21], [22], [23], [24]. For this, pulsed near-infrared laser light is rapidly emitted in targeted tissues causing thermoelastic expansion of molecules, which then generates detectable ultrasonic signals [21]. We present the first diagnostic case-control proof-of-concept study for handheld MSOT imaging to visualize and quantify muscle changes and disease burden in pediatric patients with SMA type I-III.

## 2 Materials and methods

### 2.1 Study design

The investigator-initiated (IIT) study was approved by the local ethics committee at the University Hospital Erlangen, Germany (reference: 168_19B) and conducted according to the provisions of the Declaration of Helsinki. Trial Registration: NCT 04115475. All parents or legal guardians of eligible children provided written informed consent before participation and their children provided assent as appropriate, based on the child’s age.

### 2.2 Subjects

10 pediatric patients with SMA and 10 gender and age-matched healthy volunteers were recruited at the Department of Pediatric Neurology at the University Hospital Erlangen. Key eligibility criteria for infantile SMA patients were genetically documented 5q SMA type I, II or III independent from SMN2 copy number. For HV any anamnestic suggested myopathies, and for both HV and SMA patients, pregnancy and skin tattooing at the imaging site were defined as exclusion criteria.

### 2.3 Study flow

After checking inclusion and exclusion criteria, clinical motor function tests according to age and current best motor milestones of the participant were assessed (see Supplementary Table 1). Subsequently, regular B-mode ultrasound and MSOT imaging were performed on predefined muscles on eight anatomical muscle sites in standardized transversal imaging planes: upper arm (biceps muscle), lower arm (forearm flexors), upper leg (quadriceps muscle) and lower leg (triceps surae muscle) on both sides, respectively. The exact positioning was determined using common anatomical landmarks (biceps muscle: 2/3 between acromion and cubital fossa; flexor muscles: 1/3 between medial epicondyle and thumb base, supinated; quadriceps muscle 1/2 between inguinal ligament and tip of patella in a sitting position; triceps surae muscle: 1/3 of popliteal fossa and middle malleolus) [22], [25]. For coupling transparent ultrasound gel was used (“Ultraschallgel”, medimex GmbH, Limburg, Germany or AQUASONIC clear®, Parker Laboratories Inc., Fairfield, NJ, USA). Two transversal MSOT scans were performed per muscle site.

### 2.4 B-mode ultrasound details

For all B-mode ultrasound examinations a single high-end portable ultrasound system was used (Mindray, Zonare ZS 3, Zonare Medical System Inc, Mountain View, CA Linear probe L14–5w, 12 MHz) by a single professional investigator (JJ, German Society for Ultrasound in Medicine (DEGUM) level III certified sonographer/physician). The investigator assessed echogenicity (hypoechogenic/echogenic/hyper-echogenic), muscle texture (coarse-/medium-/fine-granular), distribution pattern (inhomo-/homo-geneous/focal) and Heckmatt scale (grade 1–4: 1 = normal muscle echo, 2 = increased muscle echo while bone echo is still distinct, 3 = increased muscle echo and reduced bone echo, 4 = very strong muscle echo and complete loss of bone echo) in parallel to the examination [22], [26], [27]. Furthermore, the muscles overall impression was classified as healthy or pathological by the investigator.

### 2.5 MSOT technical details

A prototype hybrid ultrasound (Reflected ultrasound computed tomography (RUCT))/MSOT imaging system (MSOT Acuity Echo, iThera Medical GmbH, Munich, Germany). The laser operated at a repetition rate of 25 Hz and MSOT images were obtained at 680 nm, 715 nm, 730 nm, 760 nm, 800 nm, 850 nm, 930 nm, 950 nm, 980 nm, 1000 nm, 1030 nm, 1064 nm and 1100 nm. As to manufactures information, the maximum permissible exposure (MPE) of delivered energy was below legal requirements. A selective frame averaging algorithm was used to average the frames of the same wavelength from 7 consecutive wavelength cycles. For single wavelengths, the frame rate was 0.28 s/frame; for multispectral images, the frame rate was 3.64 s/frame. For all studies a handheld 2D probe (4 MHz, 256 transducer elements, field of view 40 x 40 mm, spatial resolution 150 µm) was used. Coupled by transparent ultrasound gel, the detector probe was positioned at about 90 degree angle on the skin, creating reflected-ultrasound computed tomography (RUCT) images for muscular guidance simultaneously with real-time MSOT images. The software provided a motion indicator based on the selective frame averaging algorithm, so that no motion compensation was required. For laser safety, all patients and examiners wore safety goggles.

### 2.6 Data analysis

After MSOT image acquisition was completed in all participants, data was transferred to a workstation and analysis was performed using cLabs software (V2.65, iThera Medical GmbH, Munich, Germany). For analyses, an independent blinded reader (VD) traced a polygonal region of interest (ROI) just beneath the muscle fascia according to the RUCT image. The content of the ROI was finally used for analysis of single-wave length (SWL) MSOT values as well as MSOT parameters deoxygenated hemoglobin (Hb), oxygenated hemoglobin (HbO2), collagen and lipid. A linear regression algorithm was used for spectral unmixing. MSOT parameters were spectrally unmixed [21], [28] using 715 nm, 730 nm, 760 nm, 800 nm and 850 nm for Hb and HbO2 and all SWLs for collagen and lipid. All MSOT signals are given in arbitrary units (a.u.).

### 2.7 Device tolerability

All subjects and parents/guardians were interviewed and the skin was checked about any concerns during and after the study. All complaints were documented.

### 2.8 Statistical analysis

This study was designed as a pilot trial with no sample size calculation as no information on the expected group differences were available. Descriptive data are given as mean and standard deviation [SD] or numbers and percentages. To assess homogeneity between scans (scan 1 vs. scan 2) the intraclass correlation coefficient (ICC) was calculated using the model two-way mixed effects, absolute agreement, single measurement [29], [30], grading by Landis and Koch [31].

Prior to further statistical analysis Shapiro-Wilk test was used to test the data for normal distribution. Normal distributed data was than compared by dependent samples *t*-test. In cases of not normal distributed data, a non-parametric statistical hypothesis test (Wilcoxon signed-rank tests) was used to compare the groups.

Likewise, correlation coefficients are given as Pearson (r) or Spearman (r_s_). Receiver operator characteristics (ROC) analysis between HVs and SMA-patients are shown using the genetically determined diagnosis as basis for the analyses.

For the comparison between SMA types, due to the very limited sample size, non-parametric Kruskal-Wallis test was used, a test for comparison between more than two groups. For the comparison between pharmacologically treated and not treated patients independent samples *t*-test was applied.

To increase reliability of the samples *t*-test in cases of unequal variances, Welch’s correction was used.

All tests were two-tailed and statistical significance was indicated by p values ≤ 0.05. For all analysis GraphPad Prism (Version 8 or newer, GraphPad Software, La Jolla, CA, USA) or IBM SPSS Statistics (Version 24 or newer, IBM Corporation, New Orchard Road, Armonk, NY, USA) were used.

## 3 Results

### 3.1 Patients’ characteristics

10 healthy volunteers (HV) were gender and age matched to 10 SMA patients. All participants were investigated between November 11, 2019 and January 30, 2020. The mean [SD] age was 8.7 [4.3] years in HV compared to 9.0 [3.7] years in SMA patients’ cohort. In each group, 3 [30%] subjects were females. Five [50%] of the SMA patients were treated with Nusinersen (Spinraza®, Biogen). Two patients were diagnosed SMA I, four SMA II, and four SMA III. Further patient details are presented in Table 1. The duration for each examination is presented in Supplementary Table 2**.**

**Table 1:** Characteristics of SMA-patients and healthy volunteers (HV).

Characteristics	HV (n = 10)	SMA (n = 10)
age – yr	8.7 ± 4.3	9.0 ± 3.7
age – mo	109.3 ± 53.4	114.3 ± 44.3
Female sex – no (%)	3 (30%)	3 (30%)
height – cm	138.2 ± 24.4	133.2 ± 20.4
weight – kg	33.8 ± 17.4	30.5 ± 15.5
BMI – score	16.3 ± 3.2	16.3 ± 5.4
SMA-patients specific characteristics		
Gastrointestinal tube – no (%)		1 (10%)
Ventilator support – no (%)		2 (20%)
Only during night – no (%)		1 (10%)
Long-term medication-no.(%)		
Nusinersen		5 (50%)
No specific SMA medication		5 (50%)

Yr = years, mo = month, no. = number of subjects, HV = healthy volunteers, SMA = SMA patients, Mean and standard deviation (SD) are labeled plus-minus ( ± ) values. n = 20 biologically independent subjects (n = 10 HV / n = 10 SMA patients).

### 3.2 Clinical standard assessment

All 10 HV completed the Hammersmith functional motor scale-expended (HFMSE), Revised upper Limb Module (RULM) and 6-minute-walk-test (6-MWT). 9 SMA patients performed the HFMSE and RULM (one patient was not able to follow instructions due to mental retardation; The Hammersmith Infant Neurological Examination, Section 2 (HINE) and The Children’s Hospital of Philadelphia Infant Test of Neuromuscular Disorders (CHOP Intend) were completed in this patient). 3 SMA patients performed HINE and 2 patients CHOP Intend. 2 SMA patients were able to perform the 6MWT. Overall scores were significantly lower in SMA patients (matched n = 9 HV vs. n = 9 SMA patients: HFMSE score [SD]: 65.6 [1.0] vs. 25.7 [21.8], p = 0.0039; RULM score [SD]: 36.8 [0.4] vs. 24.0 [10.5], p = 0.0156) (Supplementary 3).

### 3.3 B-mode ultrasound

A total of 160 independent muscles of HV (n = 80) and SMA patients (n = 80) were evaluated. In HV all 80 (100%) muscles were rated normal. In contrast 72 (90%) independent muscles of the SMA patients showed overall pathologic rating (Supplementary Table 4).

### 3.4 Optoacoustic imaging approach

**Fig. 1:**
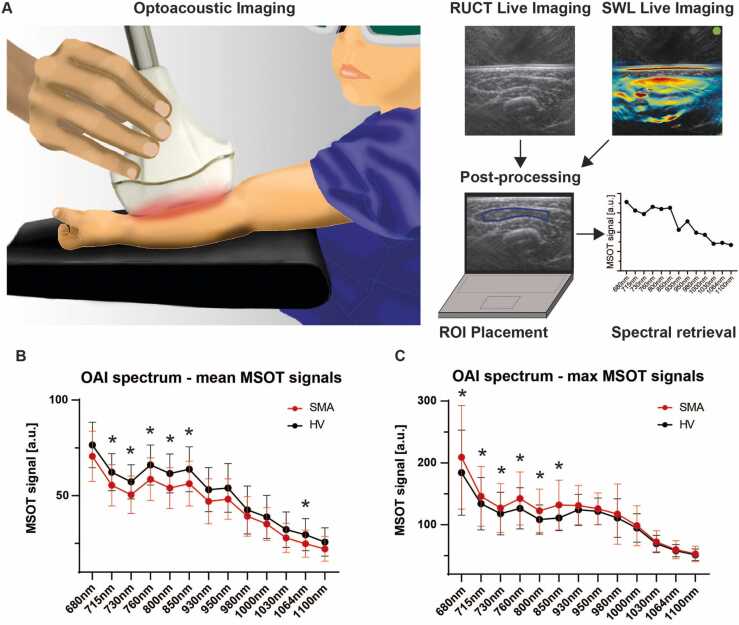
MSOT principle and optoacoustic spectrum differences between groups. a) The cartoon visualizes real-time MSOT imaging in pediatrics. Patients are placed in a relaxed position only wearing safety googles for eye protection during imaging. The investigator is guided by live reflected ultrasound computed tomography (RUCT) images and optoacoustic images (e.g. SWL 800 nm), in parallel. Raw data is than analysed after ROI placement followed by signal quantification for spectral retrieval by means of single wavelength intensities. b) The mean optoacoustic signal within the ROI is shown for n = 59 matched independent muscle regions for SWL 680 nm, 715 nm, 730 nm, 760 nm, 800 nm, 850 nm, 930 nm, 950 nm, 980 nm, 1000 nm, 1030 nm, 1064 nm and1100nm. Shown are mean [SD] signal intensities in arbitrary units (a.u.). n = 59 pairs (n = 59 independent muscles of n = 10 HV and n = 59 independent muscles of n = 10 SMA patients. Statistically significant differences are marked with a star (*) with detailed signal intensities and p values described in the main text, paired *t*-Test or Wilcoxon-sign-rank test, as appropriate. c) The maximum optoacoustic signal within the ROI is shown for n = 59 matched independent muscle regions for SWL 680 nm, 715 nm, 730 nm, 760 nm, 800 nm, 850 nm, 930 nm, 950 nm, 980 nm, 1000 nm, 1030 nm, 1064 nm and1100nm. Mean [SD] signal intensities in arbitrary units (a.u.). n = 59 pairs (n = 59 independent muscles of n = 10 HV and n = 59 independent muscles of n = 10 SMA patients. Statistically significant differences are marked with a star (*) with detailed signal intensities and p values described in the main text, paired *t*-Test or Wilcoxon-sign-rank test, as appropriate, p < 0.05 was regarded statistically significant.

After non-invasive MSOT imaging was completed, data was post-processed and two independent scans of each muscle were analyzed. A schematic overview of pediatric MSOT imaging is presented in Fig. 1a.

In total, 320 scans (160 of HV, 160 of SMA) were evaluated. 50 scans (8 scans of 1 HV, 42 scans of 5 SMA patients) did not show sufficient optoacoustic signals within the ROI, most likely due to muscle depth and were therefore excluded (mean [SD] depth of included (n = 270) vs. excluded (n = 50) scans was 7.8 [2.3] mm vs. 16.6 [3.3] mm, p < 0.0001). Of the excluded scans, 48 scans were lower and 2 were upper extremity. The remaining scans (152 of HV, 118 of SMA patients) were further statistically analyzed. First, optoacoustic signals (SWL 800 nm, MSOT parameters HbT, collagen, and lipid) were compared between scan 1 and scan 2. ICC was excellent for SWL 800 nm (ICC 0.95, 95% CI 0.91–0.97), MSOT parameter collagen (ICC 0.93, 95% CI 0.89–0.96) and MSOT parameter lipid (ICC 0.91, 95% 0.87–0.93) and good for MSOT parameter HbT (ICC 0.66, 95% CI 0.56–0.75). For further analysis, mean values of scan 1 and scan 2 were used.

### 3.5 Imaging phenotyping of healthy and diseased muscles

The primary endpoint was the comparison of the optoacoustic spectrum between HV and SMA patients by means of single wavelength (SWL) intensities. In respect to the heterogeneity of the distribution pattern of the disease, each muscle was considered independently. 21 muscle regions were excluded as described above and the remaining 59 matched muscle regions were compared. The spectrum derived from 13 SWL showed overall higher mean signals values in HV compared to SMA patients, with statistically significant differences for: MSOT SWL ( in a.u.) 715 nm (mean [SD], 59.5 [7.9] vs. 55.4 [10.8], p = 0.0274), 730 nm (54.8 [7.4] vs. 50.4 [9.8], p = 0.0133), 760 nm (62.8 [8.0] vs. 58.6 [11.1], p = 0.0261), 800 nm (58.6 [7.7] vs. 54.0 [10.7], p = 0.0082), 850 nm (60.3 [8.6] vs. 56.2 [11.7], p = 0.0391), and 1064 (27.8 [7.5] vs. 24.8 [7.0], p = 0.0387). Overall, the greatest difference of mean signal intensity was observed for SWL 800 nm (Fig. 1b).

**Fig. 2:**
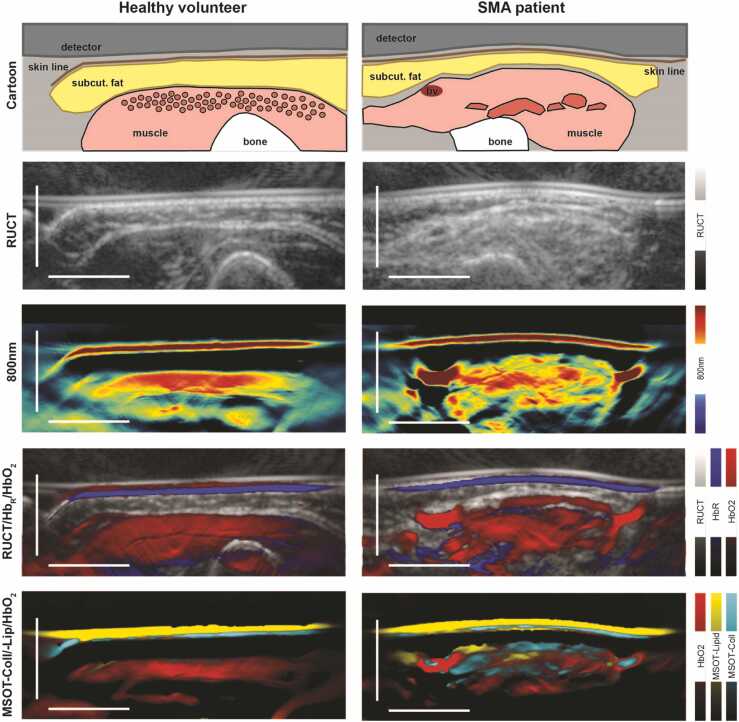
MSOT imaging of healthy and affected muscles. To illustrate differences between healthy and affected muscles, exemplary image sections of the right biceps from a SMA patient and the respective HV are shown. From top to bottom: a schematic cartoon, reflected ultrasound computed tomography (RUCT) images, MSOT SWL 800 nm, MSOT parameters HbR, HbO2, collagen and lipid images are displayed. The cartoon shows the muscle within its margins. In HV homogenous muscle fibers are expected, whereas patchy clusters of hypertrophic and atrophic muscles fibers lead to the moth-eaten pattern in SMA patients. RUCT images show inhomogeneous muscle tissue, with increased echogenicity of the diseased muscle. Optoacoustic SWL 800 nm images reveal patchy signal patterns with alternating very high and very low signal intensities in SMA patients in contrast to a homogenous signal band in the healthy muscle. Merged images of different MSOT parameters (HbR, HbO2, collagen, lipid) suppose patchy clusters of increased fatty and fibrotic transformed tissue components. Scale bar indicates 1 cm. bv = blood vessel, subcut. fat = subcutaneous fat.

In contrast, the spectrum of maximum (max) values was higher in SMA patients, with statistically significant differences for SWL (in a.u.) 680 nm (mean [SD], 174.3 [54.8] vs. 209.1 [83.6], p = 0.0104), 715 nm (127.3 [32.0] vs. 145.9 [48.2], p = 0.0092), 730 nm (112.6 [26.2] vs. 127.0 [39.4], p = 0.0148), 760 nm (120.3 [24.6] vs. 142.4 [42.8], p = 0.0005), 800 nm (103.5 [17.0] vs. 122.6 [35.0], p = 0.0002), and 850 nm (106.4 [15.3] vs. 132.0 [40.2], p < 0.0001), respectively (Fig. 1c). For better visualization of differences for SWL 800 nm and unmixed MSOT parameters (hemoglobin, collagen, lipid) between muscles of HV and SMA patients’ photoacoustic images are presented in Fig. 2. As a main finding, optoacoustic imaging (OAI) signals (see SWL 800 nm) in healthy muscles showed a homogenous signal band just beneath the muscle fascia. In muscles of SMA patients, OAI signals were found ragged in patchy scattered areas comparable to moth-eaten damage with alternating high and low signal intensities (Fig. 2). For signal quantification, the defined ROIs included both, areas with high and low OAI signals.

The spectrally unmixed MSOT parameters were compared for every muscle region (matched 59 scans) between HV and SMA patients. Mean MSOT signals of HbR (mean [SD], 41.7 [9.1] vs. 44.3 [13.5], p = 0.38), HbO2 (34.3 [6.9] vs. 34.5 [9.9], p = 0.88), HbT (76.1 [14.9] vs. 78.9 [21.9], p = 0.62), and Lipid (6.6 [4.3] vs. 7.1 [5.7], p = 0.97) did not show statistically significant differences, while MSOT collagen was higher in HV (13.1 [5.5] vs. 10.8 [6.0], p = 0.0405). Considering the maximum MSOT signal level, MSOT parameter lipid was higher in SMA patients (40.1 [18.1] vs. 53.0 [26.5], p = 0.0050), whereas MSOT parameter collagen was not significantly different (41.7 [10.7] vs. 46.0 [16.0], p = 0.08).

### 3.6 Correlation of imaging and clinical standard assessments

**Fig. 3:**
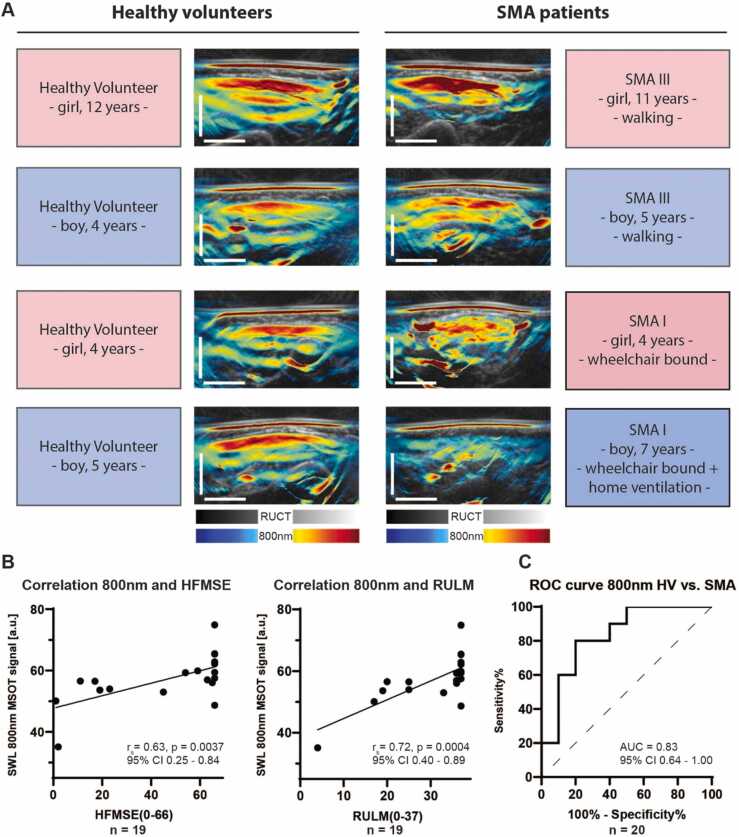
MSOT imaging and correlation with disease burden and clinical standard assessment. a) Exemplary image sections of the right biceps of four SMA patients with their matched HVs are shown to illustrate differences between MSOT SWL 800 nm signal intensities. In HVs a homogeneous signal band was detected just beneath the muscle fascia independent of gender and age. In SMA III patients one saw beginning changes towards inhomogeneous signal intensities in the muscles. In SMA I patients signal intensities were patchy transformed or even (finally) erased. Scale bar indicates 1 cm. b) Correlations between clinical standard assessments (HFMSE, score 0–66; RULM, score 0–37) and MSOT SWL 800 nm (signal intensity in a.u.) are shown for n = 19 participants. Spearman correlation coefficient (r_s_), a p value ≤ 0.05 was regarded as statistically significant. c) A receiver operator characteristics (ROC) analysis was calculated for pooled MSOT SWL 800 nm (signal intensity in a.u.) between HVs and SMA-patients (n = 10 HV and n = 10 SMA patients).

The heterogeneity of the SMA study population required correlation of individual disease status and respective MSOT signals. Best optoacoustic signal difference between both groups (HV vs. SMA) was quantified and visualized for SWL 800 nm. Based on this, optoacoustic images stratified for different SMA severity types, age and gender are presented in Fig. 3a. While HV show a homogenous signal band beneath the muscle fascia, the muscle pattern starts to be disrupted in SMA patients with preserved walking ability. The more SMA patients are affected, the more is the optoacoustic signal scattered or even (finally) erased (Fig. 3a).

To eliminate potential confounding errors, HV data were analyzed for gender (pooled MSOT SWL 800 signal for each participant, n = 3 girls vs. n = 7 boys, 59.7 [3.0] vs. 61.5 [8.4], p = 0.75) or age (pooled MSOT SWL 800 signal for each participant correlated with age, n = 10, r = 0.001, p > 0.99) specific differences which could not be observed. In this context, no correlation between SWL 800 nm signal and muscle depth (depth of ROI placement) was found in neither group (n = 76 muscles in HV, r_s_ = −0.11, p = 0.37; n = 59 muscles in SMA patients, r = −0.15, p = 0.25).

For the correlation of MSOT signals and quantitative motor outcome measures (HFMSE, RULM), a pooled MSOT signal value for each participant was used. Pearson correlation coefficient was calculated between SWL 800 nm, MSOT parameters HbR, HbO2, HbT, collagen_mean/max_, lipid_mean/max_, and HFMSE score (0–66 points) as well as RULM score (0–37 points). MSOT SWL 800 nm showed good correlation with both, HFMSE (n = 19, r = 0.63, p = 0.0037, Fig. 3b) and RULM scores (r = 0.72, p = 0.0004, Fig. 3b) and a high Area under the curve (AUC) (0.83, 95% CI 0.64–1.00) to distinguish between HV and SMA patients (Fig. 3c). In contrast, most MSOT parameters showed no significant correlation to clinical scores (n = 19, HFMSE correlated with HbR, HbO2, HbT, lipid_mean_, collagen_max_, and lipid_max_, r = 0.08, 0.31, 0.21, 0.23, −0.12 and −0.34, all p > 0.05, respectively; n = 19, RULM correlated with HbR, HbT, lipid_mean_, collagen_max_, lipid_max_, r = 0.34, 0.44, 0.07, 0.03, and −0.29, all p > 0.05, respectively), with an exception for MSOT parameter collagen_mean_ (n = 19, HFMSE/RULM correlated with collagen_mean_, r = 0.55/0.49, p = 0.0151/0.0336, respectively) and the MSOT parameter HbO2 (n = 19, RULM correlated HbO2, r = 0.49, p = 0.0351).

**Fig. 4:**
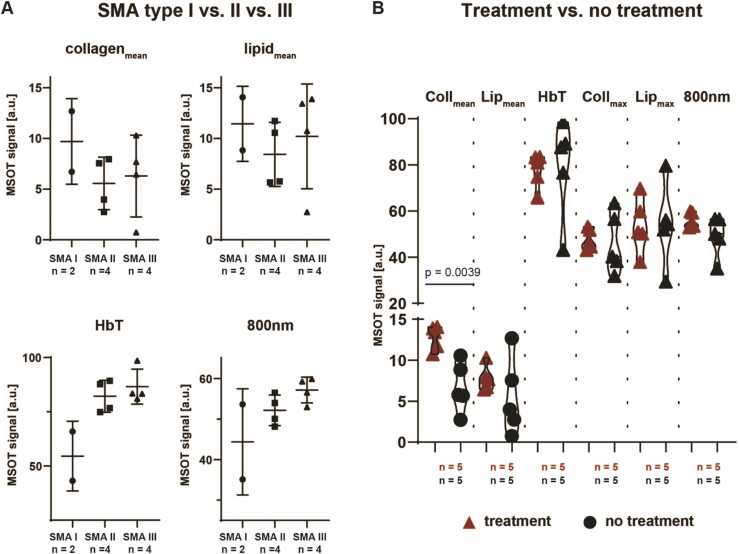
MSOT imaging and comparison of SMA subtypes/current treatment. a) MSOT parameters (collagen_mean_, lipid_mean_, HbT) and MSOT SWL 800 nm signal intensities were compared between SMA type I, II, and III using Kruskal-Wallis test, due to the very limited sample size (n = 2 SMA I, n = 4 SMA II, and n = 4 SMA III patients). p value ≤ 0.05 was regarded as statistically significant. b) Coll = collagen, Lip = lipid, MSOT parameters (coll_mean_, lip_mean_, HbT, lip_max_, coll_max_) and MSOT SWL 800 nm signal intensities were compared between SMA patients treated with and without Nusinersen using unpaired students *t*-test with Welch’s correction if appropriate (n = 5 SMA patients with and n = 5 with no treatment). The thin lines mark the density curves of the violin plot. p value ≤ 0.05 was regarded as statistically significant.

Regarding the clinical classification of SMA subtypes, SWL 800 nm showed higher values in SMA type III (n = 4) and II (n = 4) compared to SMA type I (n = 2) patients (57.2 [3.2] a.u. vs. 52.2 [3.8] a.u. vs. 44.4 [13.1] a.u., all p > 0.05). In accordance, MSOT parameter HbT showed higher values in SMA type III and SMA II compared to SMA type I patients (86.6 [8.1] a.u. vs. 82.1 [7.4] vs. 54.6 [16.1] a.u., all p > 0.05, see Fig. 4a), while MSOT parameters collagen_mean_ and lipid_mean_ did not show any trend between SMA types.

Splitting the collective in patients treated with Nusinersen (n = 5) and untreated patients (n = 5), MSOT parameter collagen_mean_ was statistically significantly higher in treated patients (mean [SD], 12.8 [1.5] a.u. vs. 6.7 [3.0] a.u., p = 0.0039) (Fig. 4b).

**Safety**. No serious adverse events occurred during the study, especially no MSOT-related adverse events (Supplementary Table 5).

## 4 Discussion

In this diagnostic proof-of-concept trial, a MSOT imaging approach demonstrated the ability to visualize and quantify progressive muscle degeneration in a heterogeneous pediatric SMA cohort when compared to healthy volunteers.

For correlation of individual disease burden and muscular function, a single wavelength approach showed the most robust results. 800 nm, the isosbestic point for imaging hemoglobin (HbO2 and HbR), was already used in various clinical applications to visualize disease features [32], [33], [34]. In this study, higher optoacoustic signals of SWL 800 nm indicated milder phenotype or higher scores in HFMSE or RULM standard clinical testing. In addition, the present study confirmed good to excellent repeatability of MSOT measurements in children, comparable to previous findings in adults [35]. This highlights the promising potential for observational or interventional longitudinal studies in children and adolescents with neuromuscular disorders [36], [37].

When using multispectral OAI systems, tissue illumination with several different SWLs and subsequent spectral unmixing of absorption patterns allows identification of single chromophores, such as hemoglobin, collagens, and lipids [22], [34], [38], [39]. While novel technical approaches in preclinical and clinical settings pave the way to new applications, the specificity to separate absorbers is still a subject of research [34], [37], [38], [40]. This might explain the finding of increased robustness of the SWL 800 nm when compared to unmixed MSOT parameters [33].

The results of this trial do differentiate from previous studies in preclinical models and patients with Duchenne muscular dystrophy (DMD), where MSOT-derived collagen parameter showed good correlation with histological findings and disease progression [22]. While dissolution of myofilaments, dilatation and focal proliferation of the sarcotubular system and fatty replacement in SMA are caused indirectly from neurogenic muscular atrophy [41], [42], [43], in DMD, the lack of a muscle structure protein directly leads to consecutive muscle fibrosis [44]. The patchy appearance with increasing loss of the optoacoustic signal depending on the severity of the SMA type can be seen as a sign of parallel existence of hypertrophic and atrophic muscle fibers. This moth-eaten pattern seen with MSOT is comparable to B-mode ultrasound [27], [45] and MRI [46], [47] findings, where ragged changes in the muscles were evident in SMA I and II, but less in SMA III patients. The fatty replacement of muscle in SMA might be reflected by the higher MSOT-derived maximum lipid levels in SMA patients compared to HV.

In contrast to other imaging modalities, MSOT has short scanning times comparable to standard ultrasound investigations [48], does not require ionizing radiation and produces quantitative measures. In turn, these modalities currently have limited penetration depths of approximately 2.5 cm from skin surface [35]. In our pediatric SMA patients no correlation between photoacoustic signal and penetration was found, which we attribute to the relatively small patient cohort. Technical improvements, such as probe optimization, increased light delivery and more sensitive detectors are required to increase penetration depth and sensitivity.

In addition, specificity of unmixing algorithms require improvements [38], [40], [49], which goes hand-in-hand with further standardization and improvement of OAI systems [50]. This, together with improved analysis and reconstruction techniques using artificial intelligence, could improve the quality of the data [51]. The use of SWL signals at the isobestic point (800 nm) showed good results in this study, but at the expense of limiting specificity and detection of other chromophores.

Furthermore, this study was limited by the small sample size, the heterogeneous patient collective and the cross-sectional design due to its pilot character. On the other hand, the broad range of SMA phenotypes in this trial underlines the ability of MSOT to assess different stages of progressive muscle atrophy and disease patterns from young age.

Especially in progressive diseases with early lethality, objective and quantitative disease monitoring approaches from birth are highly warranted. Together with the efforts towards nationwide screening [52] and disease register [53] programs for SMA, and in comparison to the current best monitoring marker of muscle testing, MSOT might open the door for a new non-invasive, bedside, non-ionizing way of early visualization and evaluation of disease burden and progression in SMA patients. As a next step, studies with larger stratified cohorts and longitudinal trials for monitoring of treatment strategies are needed.

## Funding

The project was funded by ELAN Fonds (P055) at the University Hospital of the Friedrich-Alexander-Universität (FAU) Erlangen-Nürnberg to A.P.R. F.K. and A.P.R. acknowledge support by the Interdisciplinary Center for Clinical Research (IZKF) at the University Hospital of the Friedrich-Alexander-Universität (FAU) Erlangen-Nürnberg. F.K. acknowledges founding from Else Kröner–Fresenius–Stiftung (Else Kröner-Memorial-Stipendium, 2018_EKMS.03). MJW was supported by the Graduate School in Advanced Optical Technologies of the FAU Erlangen-Nürnberg. MFN acknowledges funding from the Emerging Fields Initiative (EFI) of the FAU Erlangen-Nürnberg. M.J.W and MFN acknowledge founding from 10.13039/501100001659German Research Foundation (FOR2438, TRR241). This project has received funding from the European Union’s Horizon 2020 research and innovation programme under grant agreement No 830965. The material presented and views expressed here are the responsibility of the author(s) only. The EU Commission takes no responsibility for any use made of the information set out. HL receives support from the 10.13039/501100000024Canadian Institutes of Health Research (Foundation Grant FDN-167281), the Canadian Institutes of Health Research and Muscular Dystrophy Canada (Network Catalyst Grant for NMD4C), the 10.13039/501100000196Canada Foundation for Innovation (CFI-JELF 38412), and the Canada Research Chairs Program (Canada Research Chair in Neuromuscular Genomics and Health, 950-232279).

## CRediT authorship contribution statement

R.T., F.K. and A.P.R. conceived the idea of the study. A.P.R., A.L.W., R.T., M.J.W., and F.K. designed the study and recruited the pediatric participants. R.T., M.J.W. and F.K. were the principal investigators of the pediatric study. Ultrasound imaging was performed by J.J. MSOT imaging was performed by A.P.R., A.L.W. and F.K. Data collection was completed and analyzed by A.P.R., A.L.W., V.D. and F.K. A.P.R., A.L.W., A.F., D.K., S.S., M.F.N., H.L., J.W., R. T., M.J.W., and F.K. interpreted the data. A.P.R., A.L.W. and F.K. wrote the first draft of the manuscript. The manuscript was critically reviewed by all authors.

## Declaration of Competing Interest

The authors declare the following financial interests/personal relationships which may be considered as potential competing interests: A.P.R., M.J.W., F.K. are co-inventors together with iThera Medical GmbH, Germany on an EU patent application (EP 19 163 304.9) relating to a device and a method for analyzing optoacoustic data, an optoacoustic system and a computer program. A.P.R., M.J.W., and F.K. received travel support by iThera Medical GmbH, Germany. A.P.R. reports lecture fees from Sanofi Genzyme. F.K. reports lecture fees from Siemens Healthcare GmbH outside the submitted work. All other authors declare no competing interests.

F.K. is an editor of the Journal (“Photoacoustics”).

## Data Availability

Individual patient data will not be available for others upon reasonable request. The study protocol and statistical analysis plan are available online: clinicaltrials.gov, NCT 04115475.
