# Supplementary material for: Multispectral optoacoustic tomography for non-invasive disease phenotyping in pediatric spinal muscular atrophy patients
Source: Photoacoustics. 2021 Nov 10;25:100315. doi: 10.1016/j.pacs.2021.100315 (PMC8607197; doi:10.1016/j.pacs.2021.100315)
Supplement: Supplementary file 1 — Supplementary material [file mmc1.pdf]

**Supplementary Appendix**

This appendix has been provided by the authors to give readers additional information about their work.

Supplement to: Regensburger A. P.*, Wagner A. L.* et al. "**Multispectral Optoacoustic Tomography for non-invasive disease phenotyping in pediatric spinal muscular atrophy patients**"

**Supplementary Table 1 – Physical examination assignment**

| < 2 years | ≥ 2 years and no ability to sit | ≥ 2 years and ability to sit in wheelchair | ≥ 2 years and ability to sit | ≥ 3 years and ability to walk |
|---|---|---|---|---|
| HINE Section 2 | HINE Section 2 | HINE Section 2 | | |
| CHOP Intend | CHOP Intend | CHOP Intend | | |
| | | (HFMSE) | HFMSE | HFMSE |
| | | RULM | RULM | RULM |
| | | | | 6MWT |

**Supplementary Table 1 – Physical examination assignment**

HINE= The Hammersmith Infant Neurological Examination, Section 2 (measures motor milestones, range 0-26; lower score represents a lower development of motor milestones),[1, 2] CHOP-Intend= The Childrens's Hospital of Philadelphia Infant Test of Neuromuscular Disorders (measures motor skills, range 0-64, lower score represents a lower repertoire of motor skills),[3, 4] HFMSE= Hammersmith functional motor scale-expended (measures motor function, range 0-66, lower score represents lower motor function),[5-7] RULM= Revised upper Limb Module (measures upper limb function, range 0-37, lower score represents lower upper limb motor function),[8] 6-MWT=6-minute-walk-test (measures walking distance in meter within six minutes; range: 0-theoretically infinite; lower distance represents a higher degree of muscle function loss).[9] All subjects were tested by three well-trained physiotherapists (J.T., P.P., M. M.-A.) with respect to their age and physical function prior to ultrasound and MSOT imaging.

**Supplementary Table 2 – Duration of examinations**

| Duration of examinations | | |
|---|---|---|
| | HV (n = 10) | SMA (n = 10) |
| Duration of physical examination-min. | 32.5±6.0 | 40.3±10.1 |
| Duration of Ultrasound-min. | 10.7±2.8 | 15.1±4.5 |
| Duration of MSOT-min.* | 32.3±6.8 | 40.9±4.4 |

**Supplementary Table 2 – Duration of examinations**

Min.= minutes, HV=healthy volunteers, SMA = SMA patients, mean±SD are labeled Plus-minus values. Data are shown as mean±SD. n = 20 biologically independent subjects (n = 10 HV/n = 10 SMA). * door to door time including explanations and breaks, if necessary.

**Supplementary Table 3 – Physical examination of SMA patients and healthy volunteers**

| **Physical examination of HV and SMA patients** | | |
|---|---|---|
| | HV (n = 0) | SMA (n = 3) |
| **HINE – score** | - | 4.3±1.16 |
| | HV (n = 0) | SMA (n = 2) |
| **CHOP-Intend – score** | - | 25.0±5.7 |
| | HV (n = 10) | SMA (n = 9) |
| **HFMSE – score** | 65.6±1.0 | 25.7±21.8 |
| **RULM –score** | 36.8±0.4 | 24.0±10.5 |
| | HV (n = 10) | SMA (n = 2) |
| **6-MWT – meter** | 538.0±94.2 | 264.5±14.9 |

**Supplementary Table 3 – Physical examination of SMA patients and healthy volunteers** HV=healthy volunteers, SMA=SMA patients, mean ±SD are labeled Plus-minus values. Data are shown as mean±SD. n = 20 biologically independent subjects (n = 10 HV/n = 10 SMA). Physical tests were not uniformly completed. Incomplete tests were excluded for analysis. HINE= The Hammersmith Infant Neurological Examination, Section 2 (measures motor milestones, range 0-26; lower score represents a lower development of motor milestones),[1, 2] CHOP-Intend= The Childrens's Hospital of Philadelphia Infant of Neuromuscular Disorders (measures motor skills, range 0-64, lower score represents a lower repertoire of motor skills) [3, 4], HFMSE= Hammersmith functional motor scale-expended (measures motor function, range 0-66, lower score represents lower motor function) [5-7], RULM= Revised upper Limb Module (measures upper limb function, range 0-37, lower score represents lower upper limb motor function) [8], 6-MWT=6-minute-walk-test (measures walking distance within six minutes; range: 0-theoretically infinite; lower distance represents a higher degree of muscle function loss) [9].

**Supplementary Table 4 – B-mode ultrasound results of independent muscle regions**

| | Ultrasound Scoring | HV (N=10, N=80 scans) | SMA (N=10, N=80 scans) |
|---|---|---|---|
| **Echogenicity** | hypo-echogenic | 80 (100%) | 7 (8.75%) |
| | echogenic | 0 | 10 (12.5%) |
| | hyper-echogenic | 0 | 63 (78.75%) |
| **Muscle texture** | coarse-granular | 4 (5%) | 10 (12.5%) |
| | medium-granular | 45 (56.25%) | 0 |
| | fine-granular | 31 (38.75%) | 70 (87.75%) |
| **Distribution pattern** | Focal | 0 | 0 |
| | Inhomogeneous | 80 (100%) | 80 (100%) |
| | Homogeneous | 0 | 0 |
| **Heckmatt scale** | 1 | 80 (100%) | 13 (16.25%) |
| | 2 | 0 | 16 (20%) |
| | 3 | 0 | 34 (42.50%) |
| | 4 | 0 | 17 (21.25%) |
| **Pathological** | No | 80 (100%) | 8 (10%) |
| | Yes | 0 | 72 (90%) |

**Supplementary Table 4 – B-mode ultrasound results of independent muscle regions**

HV=healthy volunteers, SMA=SMA patients. N=160 images (n = 80 HV/ n= 80 SMA) were evaluated for echo intensity, muscle texture, distribution pattern, Heckmatt scale, and pathological rating. The investigator (JJ) assessed echogenicity (hypoechogenic/echogenic/hyperechogenic), muscle texture (coarse-/medium-/fine-granular), distribution pattern (inhomo-/homo-geneous/focal) and Heckmatt scale (grade 1-4: 1 = normal muscle echo, 2 = increased muscle echo while bone echo is still distinct, 3 = increased muscle echo and reduced bone echo, 4 = very strong muscle echo and complete loss of bone echo) in parallel to the examination.[10, 11] Furthermore, the muscle was

evaluated by the overall impression as healthy or pathological. Categorical variables are provided as numbers and percentages. n = 160 independent muscle regions (n = 80 HV/n = 80 SMA) in n = 20 biologically independent subjects (n = 10 HV/n = 10 SMA patients).

**Supplementary Table 5 – Adverse events**

| | **HV**<br>**N=10** | **SMA patients**<br>**N=10** |
|---|---|---|
| **Reversible adverse events- no. (%)** | | |
| **Coolness of Ultrasound-gel** | 1 (10%) | 1 (10%) |
| **Serious adverse events- no. (%)** | 0 (0%) | 0 (0%) |

**Supplementary Table 5 – Adverse events**

In each group one patient complained about the coolness of the ultrasound-gel. The gel was then removed. No serious adverse events occurred during the study.

*References*


1. Haataja L, Mercuri E, Regev R, et al. Optimality score for the neurologic examination of the infant at 12 and 18 months of age. J Pediatr 1999;135:153-161.
2. Bishop KM, Montes J, Finkel RS. Motor milestone assessment of infants with spinal muscular atrophy using the hammersmith infant neurological Exam-Part 2: Experience from a nusinersen clinical study. Muscle Nerve 2018;57:142-146.
3. Glanzman AM, Mazzone E, Main M, et al. The Children's Hospital of Philadelphia Infant Test of Neuromuscular Disorders (CHOP INTEND): test development and reliability. Neuromuscul Disord 2010;20:155-161.
4. Glanzman AM, McDermott MP, Montes J, et al. Validation of the Children's Hospital of Philadelphia Infant Test of Neuromuscular Disorders (CHOP INTEND). Pediatr Phys Ther 2011;23:322-326.
5. O'Hagen JM, Glanzman AM, McDermott MP, et al. An expanded version of the Hammersmith Functional Motor Scale for SMA II and III patients. Neuromuscul Disord 2007;17:693-697.
6. Glanzman AM, O'Hagen JM, McDermott MP, et al. Validation of the Expanded Hammersmith Functional Motor Scale in spinal muscular atrophy type II and III. J Child Neurol 2011;26:1499-1507.
7. Ramsey D, Scoto M, Mayhew A, et al. Revised Hammersmith Scale for spinal muscular atrophy: A SMA specific clinical outcome assessment tool. PLoS One 2017;12:e0172346.
8. Mazzone ES, Mayhew A, Montes J, et al. Revised upper limb module for spinal muscular atrophy: Development of a new module. Muscle Nerve 2017;55:869-874.
9. Montes J, McDermott MP, Martens WB, et al. Six-Minute Walk Test demonstrates motor fatigue in spinal muscular atrophy. Neurology 2010;74:833-838.
10. Heckmatt JZ, Leeman S, Dubowitz V. Ultrasound imaging in the diagnosis of muscle disease. J Pediatr 1982;101:656-660.
11. Pillen S, Arts IM, Zwarts MJ. Muscle ultrasound in neuromuscular disorders. Muscle Nerve 2008;37:679-693.
